# Myeloproliferative invasion of arterial walls: Premortem diagnosis by temporal artery biopsy

**DOI:** 10.1002/jha2.241

**Published:** 2021-06-03

**Authors:** Hannah Van Steenberge, Francesca Dedeurwaerdere, Dries Deeren

**Affiliations:** ^1^ UZ Gent, Haematology Ghent Belgium; ^2^ Laboratory Medicine AZ Delta Campus Wilgenstraat Roeselare Belgium; ^3^ AZ Delta Hospital Roeselare, Haematology Roeselare Belgium

A 72‐year old woman with a previous history of polycythemia vera presented with acute chest pain. The full blood count showed leukocytosis (WBC 43 × 10^9^/L) with 90.1% neutrophils, microcytic anemia (Hb 103 g/L), thrombocytosis (528 × 10^9^/L) and no blasts. CT scan revealed a spontaneous intramural hematoma of the descending aorta (Figure 1) and an intra‐abdominal hematoma. Active bleeding of the right internal iliac artery caused the latter and was treated with coil embolization. Bone marrow biopsy showed evolution to a myeloproliferative‐dysplastic syndrome with excessive neutrophil counts, not further classifiable. Because of the hypothesis of invasion of the vascular walls by neutrophils, we performed a biopsy of the temporal artery. Although the latter was clinically normal, the biopsy showed a dense neutrophilic infiltrate in the adventitia without inflammation or giant cells in the tunica media, not diagnostic of giant cell arteritis (Figure 2 and 3). These findings support the hypothesis of arterial wall damage and intramural hemorrhage due to infiltration of malignant neutrophils, as has been reported in some post mortem studies [1, 2]. Our case demonstrates that temporal artery biopsy can be used to safely diagnose this complication of myeloproliferative disease.

## CONFLICT OF INTEREST

The authors declare that there is no conflict of interest that could be perceived as prejudicing the impartiality of the research reported.

**FIGURE 1:**
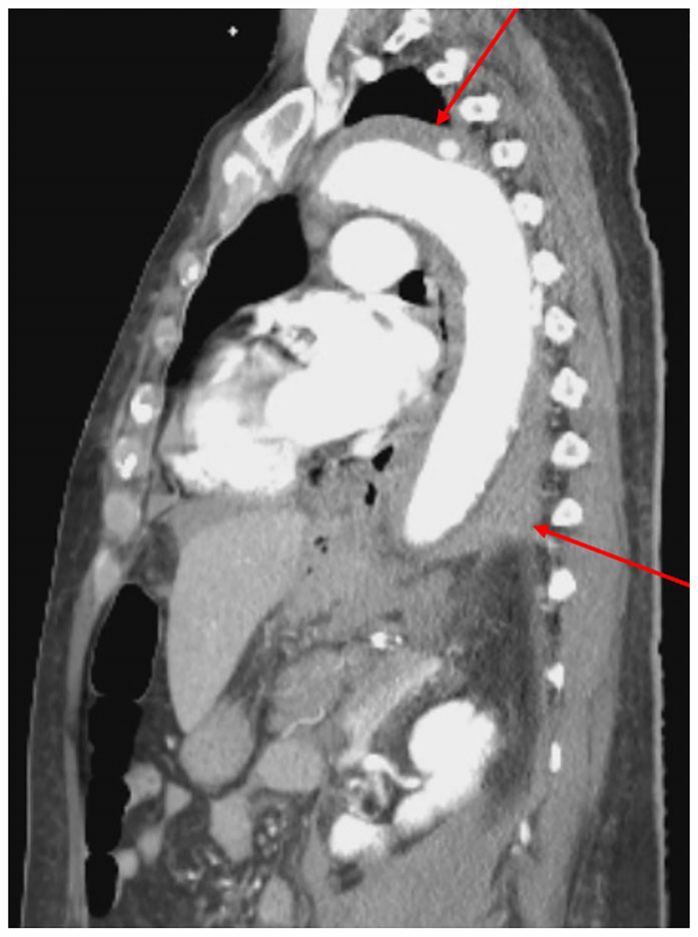
CT scan showing an intramural hematoma of the descending aorta

**FIGURE 2:**
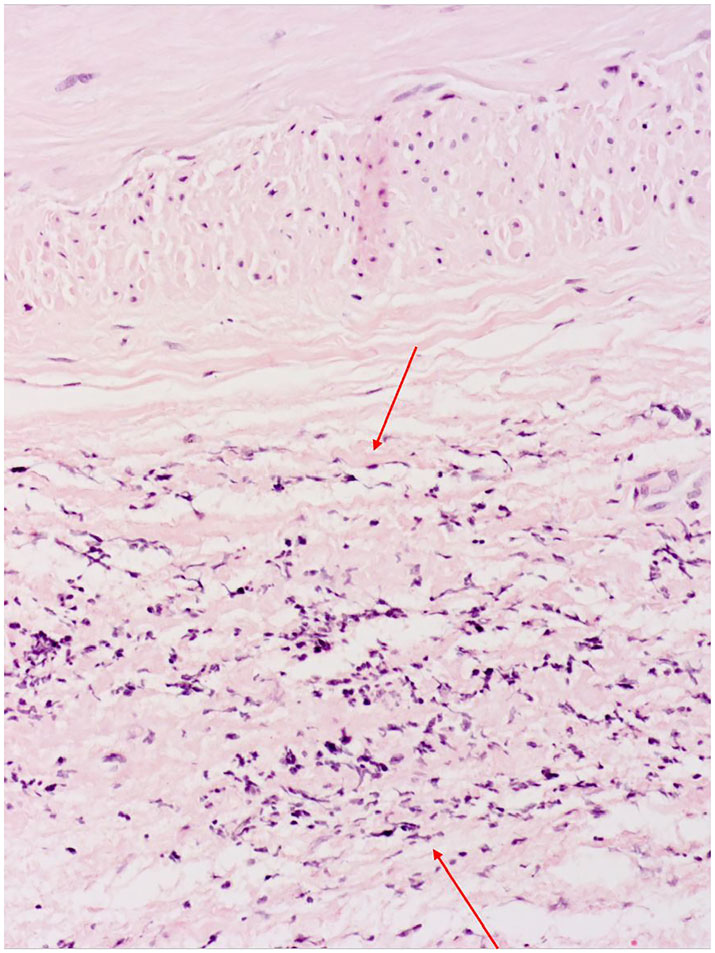
A section through a temporal artery segment (Hematoxylin eosin stain, magnification x 200) shows an increased cellularity in the tunica adventitia. The tunica intima is slightly hyperplastic, but free of inflammation. There are no signs of giant cell arteritis, as no inflammation is present in the tunica media, and no giant cells or fragmentation of the elastica interna or externa are seen

**FIGURE 3:**
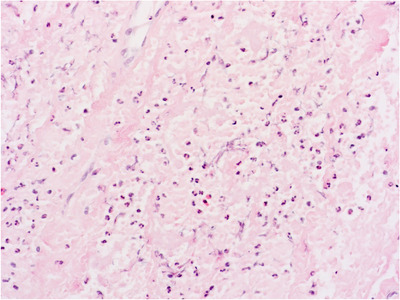
A section through a temporal artery segment (Hematoxylin eosin stain, magnification x 400). Detail of the tunica adventitia shows increased neutrophil infiltration
